# A Systematic Review on *Opuntia* (Cactaceae; Opuntioideae) Flower-Visiting Insects in the World with Emphasis on Mexico: Implications for Biodiversity Conservation

**DOI:** 10.3390/plants11010131

**Published:** 2022-01-04

**Authors:** Perla Tenorio-Escandón, Alfredo Ramírez-Hernández, Joel Flores, Jorge Juan-Vicedo, Ana Paola Martínez-Falcón

**Affiliations:** 1 CONACYT/IPICYT—División de Ciencias Ambientales, Camino a la Presa San José 2055, Colonia Lomas 4a Sección, San Luis Potosi 78216, Mexico; tenorio.perla.18@gmail.com (P.T.-E.); joel@ipicyt.edu.mx (J.F.); 2 Instituto de Investigación en Medio Ambiente y Ciencia Marina IMEDMAR, Universidad Católica de Valencia, Calle Guillem de Castro, 94, ‘San Vicente Mártir’, 46001 Valencia, Spain; jorge.juan@ucv.es; 3 Centro de Investigaciones Biológicas, Instituto de Ciencias Básicas e Ingenierías, Universidad Autónoma del Estado de Hidalgo, Carretera Pachuca-Tulancingo Km 4.5, Colonia Carboneras, Mineral de la Reforma 42184, Mexico; apmartinez@cieco.unam.mx

**Keywords:** insecta, pollination, drylands, ecological network, core species, conservation

## Abstract

*Opuntia* spp. are cacti with high ecological, economic and conservation interest in semiarid environments, particularly in Mexico. We conducted a systematic search of the existing peer-reviewed literature about the state of knowledge of pollination ecology on these plants. We documented the most studied *Opuntia* species worldwide with an emphasis on Mexico. We found that only 15% of *Opuntia* species described have been investigated so far, and studies were mainly focused on comprehension of the biology of a single species. Despite the economic and cultural importance of *Opuntia*, there is a significant lack of knowledge about the flower-visiting insects and their taxonomic identity. We provide a checklist of the insect species associated with *Opuntia* spp. Through a circular network, we visualize the complex *Opuntia* flower-visiting insect relationship, and we detected a set of key species constituting the generalist core of the networks constructed. Since pollination is crucial for crop production, a better understanding of ecological interactions would inform management measures to strengthen biodiversity and agriculture sustainability as well as productivity in arid and marginal lands. Further research on pollination ecology is needed to improve the conservation status of the insects associated with *Opuntia* species.

## 1. Introduction

Cactaceae Juss, 1789, is a highly diversified family of xerophytes that are dominant through the arid and semiarid environments of the Americas, which is its center of origin and diversification [1]. Cacti comprise approximately 1400–1800 described species in the world [2,3,4], and Mexico is the country with the greatest diversity, with 52 genera and 850 species, of which an estimated 84% are endemic [4,5]. In addition, nearly 31% of cacti are globally threatened [6] due to changes in land use; introduction of exotic species; and uncontrolled harvesting of these plants for use as food, raw material, and other purposes [7]. Some of them are listed by the International Union for the Conservation of Nature (IUCN) under various threat categories (https://www.iucnredlist.org/, accessed on 22 April 2021), which points to the need for conservation efforts.

*Opuntia* Mill. (Cactaceae; Opuntioideae) is the richest genus within the Cactaceae with nearly 200 described species [2,8,9]. *Opuntia* species are well-adapted to drought-stressed conditions [10], being highly distributed throughout arid and semi-arid environments [9]. The genus *Opuntia* is endemic to the Americas and it is distributed from Canada to Argentina [8], showing a high number of regional endemic species in Mexico [11].

However, some *Opuntia* species were introduced to other continents after the Spanish conquest [12,13,14] due to their traditional uses as medicinal plants, fruits, vegetables, dyes, food ingredients, and forage, among others [15,16,17,18].

In some cases, they have been naturalized [19,20], often resulting in problems for conservation efforts, as in Spain [20]. In some other cases, they became naturalized without any ecological problem and became a useful genetic resource with promising potential applications in cosmetics, the pharmaceutical industry, and bioenergy production [16,17]. In Mexico, the genus has a marked importance both historically and culturally because of the production of “nopal” (the Mexican denomination for the edible young cladodes developed by cacti species belonging to the genera *Opuntia* and *Nopalea*, which is translated into English as “prickly pear”) and “tuna” (Mexican name for the edible fruit of cacti species belonging to the genera *Opuntia* and *Nopalea*). In this regard, it has been estimated that the exploitation of *Opuntia* plants could generate jobs for approximately 2000 families in rural areas [21].

Asexual propagation in *Opuntia* cacti occurs naturally by stem or cladode detachment [22]. Nonetheless, sexual reproduction remains important, especially for generating viable genetic pools in wild populations, as well as for the production of “tunas” [23]. Sexual reproduction depends mainly on insects [24,25]. Therefore, improving our understanding of the relationships between *Opuntia* plants and insect pollination is critical for the conservation of both plants and insects [26,27,28] as well as for crop productivity [24].

Insect pollination has probably been crucial for the diversification and success of *Opuntia* in colonizing the American continent [1,23]. Bees in particular have been widely recognized as the main pollinators of *Opuntia* species [29], where mellitophilous syndrome dominates [25]. Visitation by insects depends on the color and scent of the flower (which is usually hermaphroditic), taste and quality of the nectar, and pollen production [30], as well as on the morphology of the visitors and the spatiotemporal abundance of both flowers and flower visitors [31].

Although some *Opuntia* species could be visited by birds such as hummingbirds (see Pimienta and del Castillo [32]), the most dominant taxonomic group of pollinators are the insects. As the global entomofauna is highly diversified, including more than one million described species, and it is broadly distributed, the aim of this study was to conduct an exhaustive and systematic review of the existing peer-reviewed literature on the species richness and composition of flower-visiting insects associated with *Opuntia* species. We documented the most studied *Opuntia* species worldwide, with an emphasis on Mexico, and provide a checklist of the insect species associated with the flowers of these species. Moreover, we visualized the complex *Opuntia*–flower-visiting-insect relationship by building a qualitative circular ecological network. Ecological networks are very useful to the study of interactions between flower-visiting insects and plant species because they provide a visual representation of community structure [33]. They also offer a broad overview of the number of interactions between species, with generalist organisms having a wide number of interactions (=core species) and other organisms having few connections with the generalists (=periphery species) [34].

## 2. Results

A total of 5406 sources of literature were found through the three academic databases examined. After the removal of literature not related to flower-visiting insect species and duplicated papers, the final number of studies was reduced to 33 articles to be analyzed. However, only 29 articles were worked on (Table S1), because two of them gave no evidence that the capture of insects was directly on the *Opuntia* flowers [35,36]. The screening process is described in the Supporting Material (Figure S1).

**Figure 1:**
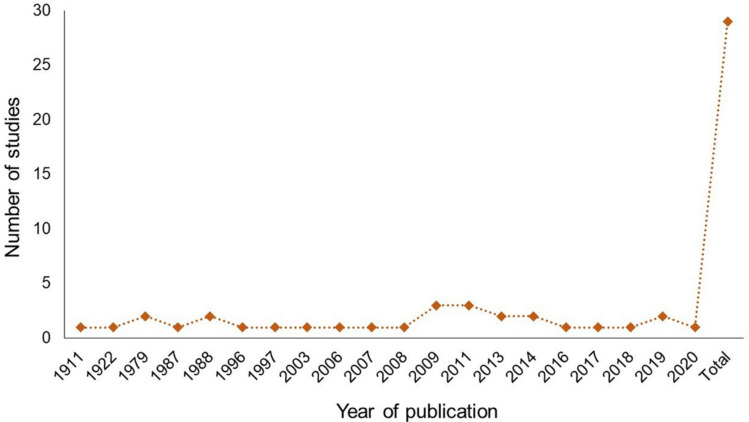
Number of retrieved articles from search in three academic databases about flower-visiting insects associated with *Opuntia* species, published from 1911 to 2020 worldwide. Note the discontinuity in the years of publication.

We found that the publication date of articles ranged from 1911 to 2020 worldwide, with 2009 and 2011 being the years with the largest number of studies (Figure 1; Table S1). In Mexico, papers were obtained from the years 1988 to 2019 [32,37,38,39,40,41,42,43,44].

**Figure 2:**
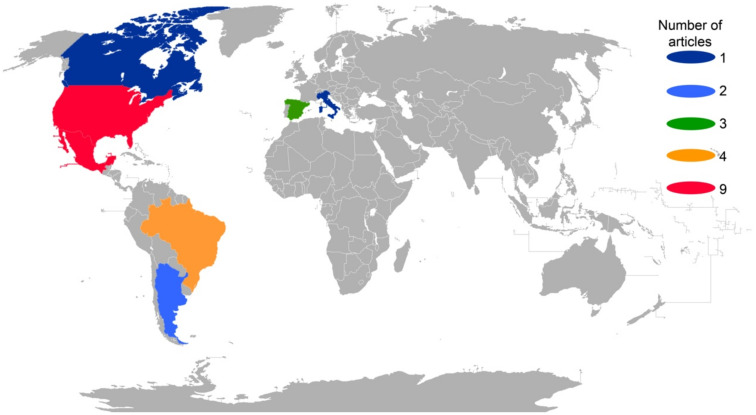
Number of retrieved studies published between 1911 and 2020 related to flower-visiting insects in *Opuntia* spp. by country.

When we analyzed the geographical component of the literature checked, we found that 25 articles were about studies carried out in the American continent, and only four studies were carried out in Europe (Figure 2). Moreover, our results show that the highest number of publications was in North America (19), particularly the U.S.A. and Mexico (Figure 2; Table S1).

**Figure 3:**
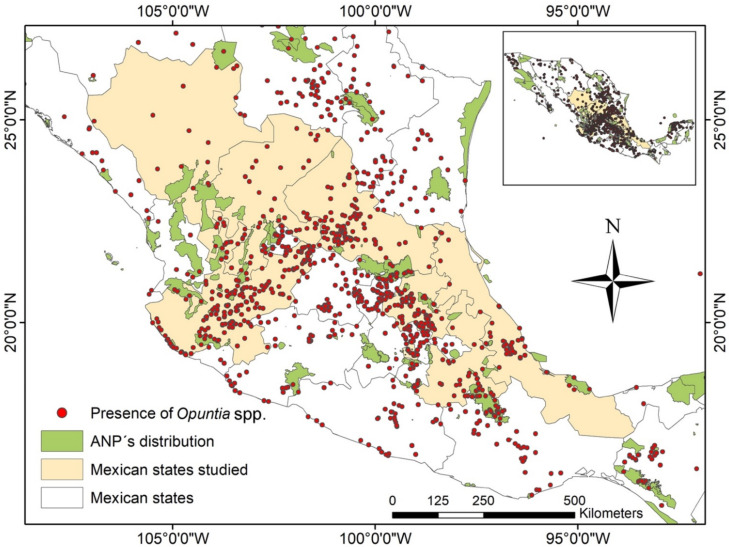
Distribution of *Opuntia* species throughout Mexico, highlighting the states where studies in articles retrieved were carried out, as extracted from the Global Biodiversity Information (GBIF).

With regard to Mexico, we found only nine studies (Figure 2) in seven states of the country (Figure 3): Mexico City [39], Durango [38,41], Hidalgo [42], Jalisco [40], Puebla [32], San Luis Potosí [37], Veracruz [43], Jalisco, and Zacatecas [44].

Of the *Opuntia* species investigated so far, an overall number of 29 taxa have been reported in the context of flower-visiting insects worldwide. We found that 21 species were studied in Canada, the U.S.A., Brazil, Argentina, Spain, and Italy (Table S1); only nine species were studied in Mexico (Table S1): *Opuntia ficus-indica* (L.) Mill., 1768, *O. huajuapensis* Bravo, *O. microdasys* (Lehm.) Pfeiff., *O. pilifera* F.A.C. Weber, *O. rastrera* F.A.C. Weber, *O. robusta* J.C. Wendl., *O. spinulifera* Salm-Dyck, *O. streptacantha* Lem. and *O. tomentosa* Salm-Dyck.

**Figure 4:**
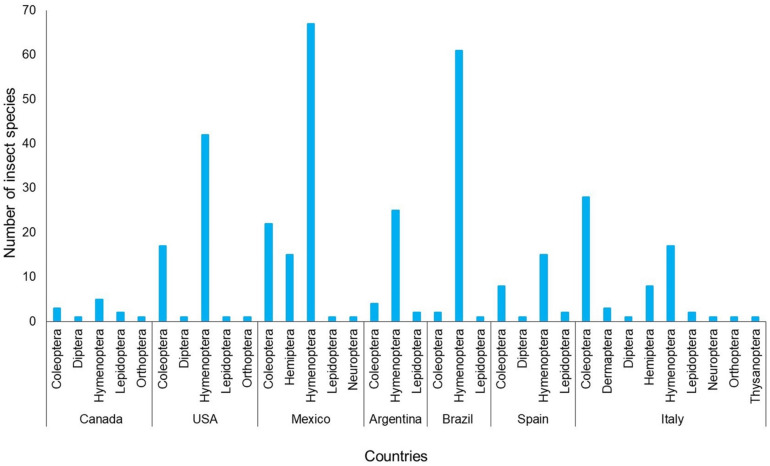
Number of orders of insect species associated with the flowers of *Opuntia* species in retrieved articles found through three academic databases between 1911 and 2020 worldwide. Further details in Table S1.

As far as insect diversity is concerned, a total of nine orders of insects were identified as *Opuntia* flower-visiting species around the world: Hymenoptera, Coleoptera, Lepidoptera, Diptera, Orthoptera, Hemiptera, Neuroptera, Dermaptera, and Thysanoptera. The results showed that Hymenoptera species were the most reported taxa among the articles published so far. Overall, a total of 318 insect species were flower visitors associated with *Opuntia* worldwide (Figure 4; Table S1).

**Figure 5:**
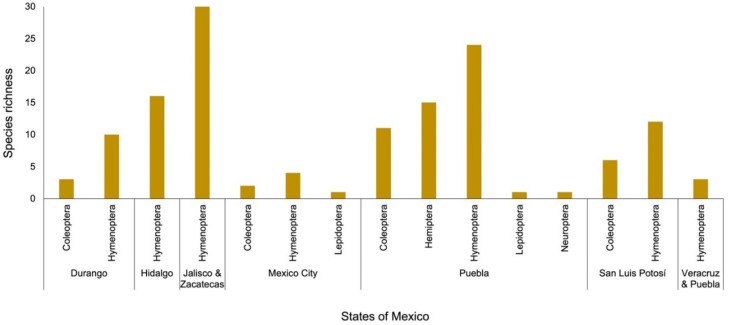
Number of insect species in each order that have been recorded as associated with flowers of *Opuntia* species in retrieved articles published in academic databases, which were organized alphabetically by states in Mexico. Further details are shown in Table S1.

In the case of Mexico, five orders have been reported (Figure 5; Table S1): Coleoptera, Hemiptera, Hymenoptera, Lepidoptera, and Neuroptera. A total of 106 species of insects have been listed. Puebla was the state with the highest number of orders and insect species recorded. Veracruz was the state with the lowest number of species of the Hymenoptera order. In Mexico, *O. pilifera* was registered with the highest number of flower-visiting insect species, which was followed by *O. ficus-indica* (Figure 5; Table S1).

**Figure 6:**
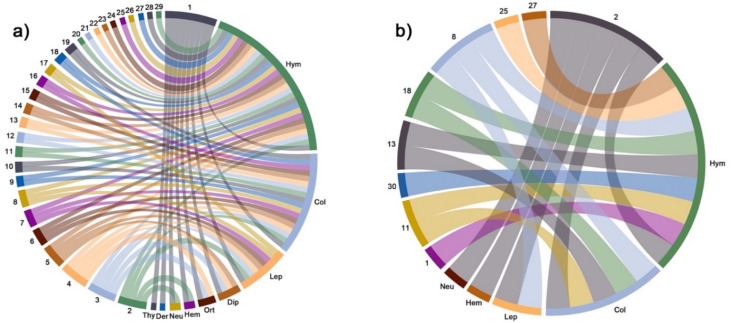
Circular network configuration of flower-visiting orders of insects associated with *Opuntia* species constructed with the databases of the retrieved articles between 1911 and 2020. Databases were sorted to construct a (**a**) global network and (**b**) network of Mexico. Numbers and abbreviations are defined as follows: Hym: Hymenoptera; Col: Coleoptera; Lep: Lepidoptera; Dip: Diptera; Ort: Orthoptera; Hem: Hemiptera; Neu: Neuroptera; Der: Dermaptera; Thy: Thysanoptera. 1: *O. ficus-indica*; 2: *O. pilifera*; 3: *O: polyacantha*; 4: *O. fragilis*; 5: *O. stricta*; 6: *O. elata*; 7: *O. maxima*; 8: *O. tomentosa*; 9: *O. monacantha*; 10: *O. lindheimeri*; 11: *O. robusta*; 12: *O. anacantha*; 13: *O. rastrera*; 14: *O. basilaris*; 15: *O. humifusa*; 16: *O. macrorhiza*; 17: *O. quimilo*; 18: *O. microdasys*; 19: *O. littoralis*; 20: *O. viridirubra*; 21: *O. spinulifera*; 22: *O. sulphurea*; 23: *O. phaeacantha*; 24: *O. retrorsa*; 25: *O. streptacantha*; 26: *O. dillenii*; 27: *O. huajuapensis*; 28: *O. macrocentra*; 29: *O. engelmannii*.

**Figure 7:**
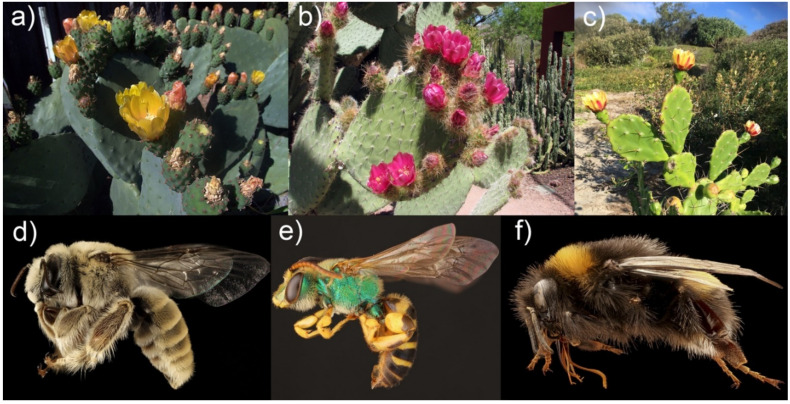
Examples of species that constituted part of the core in both global and Mexico networks. Upper figures show the flowering *Opuntia* species (**a**) *O. ficus-indica*, (**b**) *O. pilifera*, and (**c**) *O. monacantha* and lower figures show core bees (**d**) *Diadasia rinconis*, (**e**) *Agapostemon texanus*, and (**f**) *Bombus terrestris*. Note that the photos are not scaled.

The complete ecological network showed that *O. ficus-indica*, *O. pilifera,* and *O. monacantha* constituted part of the generalist core (Figure 6a and Figure 7, Table S2). The most important orders of insects were Hymenoptera, Coleoptera, and Lepidoptera. A total of 34 insect species were found constituting the generalist core of the network. In the Mexican *Opuntia*–insect network, the single species *O. pilifera* alone made up the generalist core of the network. The most important orders of insects were Hymenoptera and Coleoptera. We found nine insect species as part of the generalist core (Figure 6b and Figure 7, Table S2).

## 3. Discussion

In Mexico, *Opuntia* species represent one of the most important crops, because they cover about 30% of the country’s land area, and they are mainly distributed throughout arid and semiarid regions (Figure 3) [45]. Mexico is the main producer of prickly pears, supporting 43% of annual world production, which is estimated at 1,060,000 t in an area of 100,000 ha [46]. The area planted with nopal in Mexico in 2019 was 45,746 ha, which was mainly in the Mexican High Plateau (central Mexico).

Despite extensive research on the economic, cultural, and medicinal importance on *Opuntia* species, there are still few studies on the potential of these taxa (including crops) for the conservation of biodiversity [47]. In fact, we found that a low number of studies have been conducted on flower-visiting insect species and their relation to biodiversity conservation. This is in line with the findings by Mandujano et al. [25], who stated a decade ago that the reproductive biology of only 2% of cacti had been studied. In this review, only 29 studies provided sufficient evidence of insect capture on *Opuntia* flowers (Table S1), but the goals, period of time when the work was published, and sampling methods employed are quite heterogeneous, as we discuss below.

### 3.1. Timeline of Opuntia Studies

Since the botanical family Cactaceae is endemic to the Americas [9], it is not surprising to find a higher number of articles published on this continent, particularly in the U.S.A. and Mexico (nine articles each) (Table S1). It is evident that insect pollination attracted the interests of early researchers [48,49], who studied the main flower-visiting insects on *O. humifusa* and *O. macrorhiza*, with a taxonomic description of new bee species of the genus *Perdita* (Hymenoptera) in the U.S.A.

Further research papers were published in the 1970s that were focused on both pollination and the reproductive system of *Opuntia* spp. Grant and Grant [50] and Grant et al. [51] studied the behavior of insects and their efficiency as pollinators of *O. basilaris*, *O. littoralis*, and *O. lindheimeri*. At the end of the 1980s, new research was conducted on flower-visiting insects in San Luis Potosí in the southern Chihuahuan Desert (Mexico) [37,38].

In the twenty-first century, 18 studies have been published around the world: six in Mexico and 12 in other countries. These studies were focused on *Opuntia* biology, including pollinators [52]. Outside of the Americas, *Opuntia* species are recognized as exotic, with some considered fully naturalized, and they are often cultivated and used [53,54]. For example, *Opuntia* species have been exploited since the eighteenth century in Sicily, which is the center of cultivation of this genus in Italy [24]. Other studies addressed the invasive nature of certain *Opuntia* species, which can modify the native community structure due to their greater attraction of native insects in comparison to native plants [19,20,55].

### 3.2. Insect Diversity: Are All the Species Efficient in Pollen Transport?

The studies conducted worldwide indicate that the main flower-visiting insect species in *Opuntia* spp. are bees (Hymenoptera, Apidae), beetles (Coleoptera), and some lepidopterans (Lepidoptera). However, the effectiveness of pollinator species varied broadly. Early studies [50,51] showed that both bees and coleopterans were the main groups of insects that pollinated *Opuntia*; however, coleopteran species were not found to be efficient for pollen transport because they feed on the stamens and petals [50,51]. Therefore, the probability of pollen adhering to their bodies is very low [56]. Moreover, beetles do not fly in search of more flowers, and thus, cross-pollination between plants is limited.

In addition, the body size of insects plays a critical role in determining their effectiveness as pollinators [56]. Due to their smaller body size, bees of the genus *Perdita* sp. (2.0 mm to 10.0 mm) are not considered very effective pollinators, because they can slip into a flower without coming into contact with the stigma [51], thus limiting pollination. The recognized effective pollinators of *Opuntia* are medium and large-sized bees, such as species belonging to the genera *Diadasia*, *Lithurge*, *Melissodes*, *Bombus*, *Agapostemon*, and *Megachile* [51,57].

The few studies carried out in Argentina, Brazil and Canada were devoted to learning the structure of *Opuntia* flowers and the diversity of insects that visit them. For example, when bees touch the filaments, they stimulate the movement of sensitive stamens, causing the *Opuntia* flower to hide most of its pollen from flower visitors [23]. This floral adaptation benefits oligolectic (pollen-specialist) pollinating bees, which can reach the lower layers of the anthers, where 80% of the flower pollen is located [58]. According to the results obtained in the meta-analysis, oligolectic species (such as *Ptilothrix fructifera*, *Lithurgus rufiventris* and *Cephalocolletes rugata*) are the only effective pollinators in *Opuntia* [58,59,60]. Reliance on oligolectic bees for pollination has been similarly documented for a high number of plant species belonging to different plant families [61,62], including other Cactaceae [52,63]. Therefore, this seems to be quite likely for all *Opuntia* species as well. However, evidence in studies of other plant taxa highlights that the pollination effectiveness of different flower-visiting insects can also vary depending on other factors, such as season or geographical area, where other insects, including non-oligolectic bees, can act as effective pollinators [61]. For instance, in *Lobularia maritima* (L.) Desv. (Brassicaceae), the effectiveness of ants in pollination during the summer was comparable to the most effective insects in other periods such as spring [61]. Ants were also the main pollinators of *Jatropha curcas* L. (Euphorbiaceae) in Mediterranean croplands, unlike what is described for this species in its native distribution area [64]. As *Opuntia* spp. display a wide blooming period (ranging from early spring to the whole summer), and some of them are important crop species that are commonly grown out of their native area, it would be interesting to examine whether ants (as they are usually documented to be abundant flower visitors) or other animal species might also constitute effective pollinators in certain cases.

However, it is also important to bear in mind that ants (as occurs with other common flower-visiting insects) could not generally participate in active *Opuntia* pollination, as these plants have extrafloral nectaries (EFN). Ecologically, extrafloral nectar is important as a sugared reward for ants to assure ant protection against herbivores [65,66,67]. Having EFN, plants also prevent ants from taking nectar directly from flowers, thus ensuring pollination by other more efficient species [68] in order to achieve successful cross-pollination between different *Opuntia* individuals.

In this regard, ants could be even considered nectar thieves, because the pollen is not transported to other plant individuals as a result of their visit (mainly due to their small body size and absence of hair), and the plant does not benefit from the interaction from the sexual reproduction point of view (e.g., Komamura et al. [69]). Although there are few ant species able to transport pollen, the usual presence of metapleural gland secretion on the integument can also reduce the viability of pollen grains in these particular cases, as documented by Beattie et al. [70], Rostás et al. [71], and Rostás and Tautz [72], therefore, not contributing to the effective cross-pollination. In addition, Rostás et al. [71] found that ants visiting the flowers of *Euphorbia seguieriana* Neck. do not facilitate outcrossing because the worker ants were mainly plant dwellers; therefore, it was only flying insects that were responsible for pollination, thus increasing the real sexual reproduction measured by success in seed germination rates. Therefore, the role of ants (or other animal species) in *Opuntia* pollination beyond oligolectic bees should be carefully studied in order to achieve a better understanding of the reproductive ecology of these plants.

It is well known that cross-pollination mediated by insects in *Opuntia* is crucial for crop productivity [73]. According to Ávila-Gómez et al. [35], *Opuntia* spp. crop productivity (e.g., number of fruits) is related to the number and species composition of bees. In addition, cross-pollination maintains the genetic diversity in plant populations. Nonetheless, this issue has not been studied for *Opuntia* spp., and further research must focus on understanding how these complex interactions could support the genetic variability of *Opuntia* spp. and the impact it might have on the plant–pollinator network structure.

### 3.3. The Relationship between Opuntia and Insects in Mexico

Mexican studies focused on flower-visiting insects on *Opuntia* species have mainly been conducted in arid environments, which moreover represent nearly 50% of the country [5,74]. As noted above, these works were conducted within the Chihuahuan [37,38,40,41,47] and Tehuacán deserts [32,43], as well as in some xerophytic communities [39,42]. Only nine species of *Opuntia* have been studied in Mexico with respect to pollination biology, in spite of the great diversity that this genus displays in the country. In addition, this research was performed within the context of the economic and medicinal importance of *Opuntia* species [24,75,76] rather than conservation needs. In this regard, it would be of great interest to broaden the scope of the research beyond the usual crop *Opuntia* taxa in order to have a better understanding of the pollination ecology of species in natural environments.

The studies included in this review cover different topics ranging from *Opuntia* biology to insect interactions. For instance, del Castillo and González-Espinosa [37] studied sexual polymorphism in *O. robusta*, and they found that its flowers are mainly visited by solitary bees and coleopterans with occasional records of lepidopterans, ants, and hummingbirds.

Two studies were conducted in the Mapimi Biosphere Reserve, which is part of the Chihuahuan desert. These studies were focused on *O. rastrera* [38] and *O. microdasys* [41]; however, few records of flower visitors were obtained. In contrast, Morales-Trejo et al. [32] studied the insect diversity associated with *O. pilifera*, and they recorded the highest number of insects so far (Hymenoptera, Coleoptera, Hemiptera, Diptera, Neuroptera, and Lepidoptera were identified). Differences in the number of species recorded could be due to the different sampling methods employed. For instance, studies performed on *O. rastrera* and *O. microdasys* were focused on visual records of the frequency that insects pollinated *Opuntia* flowers. The research conducted on *O. pilifera* employed a more complete set of harvesting methods mainly consisting of direct collection using entomological nets to capture flying insects and entomological forceps for species found inside the flowers [32]. Santa Anna-Aguayo et al. [43] used a sampling method that consisted of video camera recordings in order to study the behavior of the introduced species *A. mellifera* and the native species *Lithurgus littoralis*. They highlighted possible interference by competition between native and non-native bee species that visit the flowers of *O. huajuapensis*.

The use of diverse sampling methods to study the same group of insects often produces different results. This may generate biased conclusions that in turn make it difficult to compare the diversity of species and interactions and their implications for biodiversity conservation [77]. It is difficult to standardize a single method for insect sampling, and a recommendation to achieve more complete coverage of diversity is to coordinate all data produced by different sampling approaches [78]. In this regard, assimilating information collected using multiple methods, observations, and other sources into a composite database on *Opuntia* and insect interactions would provide a basis for a better understanding of plant–insect interactions between these groups. Moreover, focusing research efforts on interactions between threatened species of *Opuntia* and insects will help the conservation and management of these species, which is currently lacking for both biological groups as revealed in this review.

### 3.4. The Role of Core Species in the Community Structure

We found that *O. ficus-indica*, *O. pilifera*, and *O. monacantha* constitute the generalist core of the *Opuntia* spp. global network, whereas only *O. pilifera* was core in the Mexican network. In a broad sense, the importance of *Opuntia* in the network can vary according to the number of studies carried out in each species as well as the sampling effort. This is probably the case for *O. ficus-indica*, which is a domesticated species with a long history of uses and has been cultivated in several countries [79,80].

For this reason, it is not surprising that *O. ficus-indica* constitutes part of the core species in the global network. *O. ficus-indica* was first introduced to Europe via Spain during the fifteenth century, and it has been cultivated in many parts of the world for various purposes, including carminic acid extraction from *Dactylopius coccus* Costa, 1835 [18,81]. In this regard, Padrón et al. [20] found that *O. maxima* is an invader species that behaves as a core species in native communities, where it becomes naturalized. It is known that flowers of alien plants are usually compatible with the body size of native insects [82]. Accordingly, these plants may attract a broad number of native insect pollinators, acting as competitors of the native flora, modifying the network’s structure and the function of the original communities because pollen transported by insects may be dominated by pollen from alien plants [54,83]. In addition, some *Opuntia* species have no limits to their pollen production, so they might sustain a vast community of insects, thus increasing seed production. Therefore, the probability that these species would expand their range of distribution is high [19].

In the Mexican network, *O. pilifera* alone constituted the core species. However, these results are probably influenced by the study conducted by Morales-Trejo et al. [32], who carried out extensive field observations with the goal of recording the highest possible number of insect species and individuals visiting the flowers of *O. pilifera* and finding seven orders of insects. They also found a temporal segregation of insects in the morning versus the afternoon. This variation can most likely be attributed to the environmental pulses that occur at different times of the day because of changes in the main abiotic factors across the day (such as humidity, temperature, solar irradiation, etc.), which can affect insect behavior and activity [84]. More comprehensive studies on the pollination ecology of *Opuntia* species from Mexico, ideally conducted with standardized insect harvesting methodologies, would be necessary to ascertain whether *O. pilifera* is still a core species in the Mexican network, as well as to elucidate the role of other *Opuntia* species as core species for this network.

In terms of the insect fauna recorded visiting *Opuntia* flowers, species belonging to the genera *Diadasia*, *Lithurge*, *Melissodes*, *Bombus*, *Agapostemon*, and *Megachile*, which were also the most effective pollinators, were core to the global network. Species of these genera have been documented as the most effective plant pollinators due to their body size [51,57]. The Eurasian honeybee, *A. mellifera*, had the highest core value. Despite the global economic importance of *A. mellifera* [85,86], it is an exotic species in the Americas [87], and its effects on the structure and functioning of native communities should also be considered. In this regard, Santa Anna-Aguayo et al. [43] found that females of *L. littoralis* avoid flowers that have been previously visited by *A. mellifera*. There is still little information about the competition effect of honeybees on native bees, and therefore, general conclusions cannot be stated. In spite of the fact that *A. mellifera* is included in the IUCN Red Lists [88,89], it could be of great interest to assess the effects of this species on the conservation of native pollinators in areas where it is not native, such as the American continent. It is important that future research prioritize the study of native bees to discover their conservation status, since competition with *A. mellifera* could be reducing their populations and affecting the reproductive capacity of *Opuntia* species.

Bumblebees (*Bombus* sp.) face important conservation challenges in North America due to their natural habitat transformation for large-scale intensive agricultural production [90,91,92] with many species included in the IUCN Red Lists. Therefore, to maintain (or recover) the conservation status and population levels of bumblebees, it could be useful to maintain the *Opuntia* species that they visit.

### 3.5. Conservation Insights on Threatened Opuntia Species

None of the *Opuntia* species found in this study were included in any Appendix of the Convention on International Trade in Endangered Species of Wild Fauna and Flora (CITES; https://cites.org/eng, accessed on 28 April 2021) nor under any category of the IUCN Red Lists of Threatened Species. Neither of the Mexican *Opuntia* species listed in this review are included in the official Mexican standard NOM-059 (SEMARNAT-2010) [93] for protection.

It is very surprising that out of the more than 30 restricted and/or endangered *Opuntia* species listed in Mexico (https://www.iucnredlist.org/, accessed on 22 April 2021), none of them were the subject of any studies on floral biology and pollination ecology (including works on flower-visiting insects), as we found in this review. For instance, the endemic Mexican species *O. chaffeyi* Britton & Rose is a Critically Endangered species with a narrow distribution restricted to three separated localities (63 km^2^) in the state of Zacatecas [94]. *O. megarhiza* Rose is endemic to the La Trinidad and Sierra de Álvarez mountain ranges in San Luis Potosí. This species has a small range area (less than 1895 km^2^) and subpopulations consisting of three disjunct subpopulations, which motivated its inclusion as Endangered by Hernández et al. [95].

Other non-Mexican *Opuntia* species that merit conservation efforts include *O. abjecta* Small ex Britton & Rose, which is listed as Critically Endangered. It occurs in three verified populations with an overall area of 65 km^2^ in Florida (U.S.A.) [96]. *O. schumannii* F.A.C. Weber ex A. Berger is listed as Vulnerable, as its presence was reported from only two locations near the Colombia–Venezuela border, where habitat loss has been described as the major threat by Nassar et al. [97]. Research to estimate the population size trends, as well as to address site protection, is needed.

Finally, some Near Threatened species are *O. triacantha* (Willd.) Sweet and *O. curassavica* (L.) Mill. The first has approximately 15 locations in a wide area with an insular distribution pattern ranging across Cuba, the Lesser Antilles, Puerto Rico, and the United States Virgin Islands. The authors point out the need for more detailed information on their current distribution, population status, and ecology [98]. *O. curassavica* has a wide distribution area (Netherlands Antilles, Colombia, Lesser Antilles, Venezuela, Curacao, Aruba, and Tortuga Island), but it is considered highly threatened as a result of the destruction of its habitat [99]. The authors also point out the need for more detailed research on this species.

An interdisciplinary strategy for *Opuntia* genus conservation, particularly in Mexico, would greatly clarify the conservation status of each *Opuntia* species and the steps needed to protect them. For this reason, further studies aimed at understanding their sexual reproduction are crucial, as asexual reproduction is not normally compromised [22]. Therefore, this makes specific studies on pollination ecology and research on the ecological networks even more important so that the interdependencies between plant and insect species may be identified, as well as possible pollination constraints that might compromise natural population viability, trends, and medium-term survival. In order to achieve this goal, it is necessary to obtain consistent field data by intensifying efforts to document the pollination ecology of these species using standardized insect sampling methods for the diverse *Opuntia* species in their native habitats across the American continent.

## 4. Materials and Methods

In order to explore the structure of species interactions between *Opuntia* and insects, a database was first produced from a systematic search of the literature using the academic databases ISI Web of Science (https://webofknowledge.com/, accessed on 12 December 2020), PubMed (https://pubmed.ncbi.nlm.nih.gov/, accessed on 13 December 2021), and Google Scholar (https://scholar.google.com/, accessed on 16 December 2021).

The literature search was carried out taking into consideration articles published up to December 2020. Filtering was applied with the purpose of compiling all studies related to flower-visiting insects in the genus *Opuntia*. Combinations of keywords used in the search were “*Opuntia*” AND “Pollination”, “*Opuntia*” AND “Floral visitors”, “*Opuntia*” AND “Insects”, “*Opuntia*” AND “Interactions”, “*Opuntia*” AND “Floral biology”, and “*Opuntia*” AND “Arthropod”.

In order to select the most suitable literature, a manual filter was applied based on the information given in the title, abstract, and keywords for all the obtained results. Only primary sources focused on *Opuntia* flower-visiting insects were used for the database, and therefore review articles, thesis, and other sources not containing additional information on this topic were purposely excluded from the database. The spelling of scientific names for plants (including authors’ abbreviations) followed the standards recommended by World Flora Online (http://www.worldfloraonline.org/, accessed on 1 April 2021). The scientific names of insects were corroborated with the help of the Global Biodiversity Information Facility (GBIF; https://www.gbif.org/, accessed on 11 April 2021). Species previously considered *Opuntia* but currently included in different genera were also excluded (for instance, *O. anteojoensis* Pinkava, which is currently ascribed to *Cylindropuntia anteojoensis* (Pinkava) E.F. Anderson; and *O. corallicola* (Small) Werderm. which is synonymized to *Consolea corallicola* Small.). Following the study of Negrón-Ortiz [100], the taxonomic identity of *O. spinosissima* Mill. was reclassified as *C. spinosissima* (Mill.) Lem (further details in Areces-Mallea [101]); the same occurred with Locatelli and Machado [102] where the taxonomic identity of *O. palmadora* Britton & Rose was reclassified as *Tacinga palmadora* (Britton & Rose) N.P. Taylor & Stuppy*,* and therefore, we excluded them from this review.

The literature retrieved from the search was compiled into a database ordered according to the year of publication, authors, and country of the study. Each *Opuntia* species studied was included with its associated insect species recorded in the papers. The current accepted names of both *Opuntia* and insect species were included in the database. Insects were sorted according to the order to which they belong.

In order to map the distribution of *Opuntia* species throughout Mexico, we constructed a map using a database downloaded from the Global Biodiversity Information Facility (GBIF) [103].

As a basis, a presence/absence dataset of insects and *Opuntia* was created using the records obtained from the retrieved papers. Then, we explored the structure of the community by building a circular qualitative ecological network in order to visualize the complex *Opuntia* flower-visiting order of insect relationships both globally and in Mexico. To visualize the network and structure of interactions between *Opuntia* species and insect orders in more depth, a presence/absence matrix was constructed where a*ij* indicates an interaction between insect species *i* and *Opuntia* species *j*, with a value of 1 when an interaction between species *i* and *j* was recorded, and 0 when no interactions were recorded [104]. Circular networks were constructed in EVERVIZ (https://app.everviz.com/create, accessed on 8 October 2021).

To understand the importance of each *Opuntia* and each insect species within the network, we performed the categorical core vs. periphery analysis that describes which species constitute the core components (generalist species, those with the most interactions) and which species are peripheral components (those with fewer interactions) of the network using the following equation [105]:

$$Gc=\frac{\left(ki\;-\;kmean\right)}{\sigma k\;}$$

where *k_i_* is the number of links for a given plant/insect species, *k_mean_* is the mean number of links for all plant/insect species in the network, and *σ_k_* is the standard deviation of the number of links for plant/insect species. Species with *Gc* > 1 are species with more interactions than other species of the same trophic level and therefore are considered species constituting the generalist core. *Gc* < 1 are species with fewer interactions than other species of the same trophic level and therefore are considered species constituting the periphery of networks.

## 5. Conclusions and Future Perspectives

Despite the historical, economical, and cultural importance of *Opuntia* species in Mexico, the scarce number of studies about the insect pollination of these species is remarkable, and research is completely lacking on *Opuntia* with conservation interests. In this review, we found that only 15% of *Opuntia* species have been investigated, and existing studies were focused on comprehension of the biology of a single cactus species (e.g., [106,107,108,109,110,111,112]), often with economic interest. There have barely been any studies with comparisons between two species. There is a bias in sampling effort and methods of studying the flower-visiting insects. Further research is needed to standardize an effective sampling protocol to monitor the broad insect diversity. Bees have been considered the main and most efficient pollinator insects, and this has probably diverted attention from other entomofauna. More research is needed to improve knowledge of the diversity of flower-visiting insects associated with *Opuntia*, especially directed toward restricted endemic, rare, or threatened plant species. These future studies should analyze the roles of all visitors in natural community maintenance and the influence of crop productivity. In addition, it is important to expand knowledge of both the influence of exotic entomofauna on native fauna, and the impact that exotic *Opuntia* can have on specialized pollinators, thereby providing us with a broader panorama of this interaction. The conservation of *Opuntia* crops and wild species not included in Red Lists can also help preserve insect species with conservation interest such as *B. pensylvanicus*. In this regard, further research on pollination ecology and network analysis is needed to improve the conservation status of the insects associated with *Opuntia*. Finally, because pollination is crucial for crop production, a better understanding of ecological interaction networks would inform management measures undertaken to strengthen biodiversity and agriculture sustainability and productivity in arid and marginal lands.

## Data Availability

Not applicable.

## Supplementary Materials

The following are available online at https://www.mdpi.com/article/10.3390/plants11010131/s1, Figure S1: Selection of studies for inclusion in the systematic review (n: the number of studies). Table S1: Checklist of the 29 Opuntia species with their associated insect flower-visiting species. This database was extracted from the 29 retrieved articles published from 1911 to 2020 worldwide. The references cited herein can be found in the References list in the main manuscript. Note that insect species are listed according to the Order and Family they belong to. Table S2: List of Opuntia and insect species that constitute part of the generalist core in both the global and Mexican networks.

## References

[1] Hernandez-Hernandez T., Brown J.W., Schlumpberger B.O., Eguiarte L.E., Magallón S. (2014). Beyond aridification: Multiple explanations for the elevated diversification of cacti in the new world Succulent Biome. New Phytol..

[2] Anderson E.F. (2001). The Cactus Family.

[3] Hunt D. (2006). The New Cactus Lexicon.

[4] Guerrero P.C., Majure L.C., Cornejo-Romero A., Hernández-Hernández T. (2019). Phylogenetic relationships and evolutionary trends in the cactus family. J. Hered..

[5] González-Medrano F. (2012). Las Zonas Áridas y Semiáridas de México y su Vegetación.

[6] Goettsch B., Hilton-Taylor C., Cruz-Piñón G., Duffy J.P., Frances A., Hernández H.M., Inger R., Pollock C., Schipper J., Superina M. (2015). High proportion of cactus species threatened with extinction. Nat. Plants.

[7] Jiménez-Sierra C.L. (2011). Las cactáceas mexicanas y los riesgos que enfrentan. Revista Digital Universitaria.

[8] Majure L.C., Puente R. (2014). Phylogenetic relationships and morphological evolution in *Opuntia* s. str. and closely related members of tribe Opuntieae. Succ. Plant Res..

[9] Majure L.C., Puente R., Griffith M.P., Judd W.S., Soltis P.S., Soltis D.E. (2012). Phylogeny of *Opuntia* s.s. (Cactaceae): Clade delineation, geographic origins, and reticulate evolution. Am. J. Bot..

[10] Aliscioni N.L., Delbón N.E., Gurvich D.E. (2021). Spine function in Cactaceae, a review. J. Prof. Assoc. Cactus.

[11] González-Elizondo M.S., González-Elizondo M., López-Enríquez I.L., Tena-Flores J.A., González-Gallegos J.G., Ruacho-González L., Melgoza-Castillo A., Villarreal-Quintanilla J.A., Estrada-Castillón A.E. (2017). Diagnóstico del conocimiento taxonómico y florístico de las plantas vasculares del norte de México. Bot. Sci..

[12] Flores Valdez C.A., Aguirre Rivera J.R. (1979). El Nopal como Forraje.

[13] Anaya-Pérez M.A., Bautista-Zane R. (2008). El nopal forrajero en México: Del siglo XVI al siglo XX. Agric. Soc. Desarro..

[14] Ortega-Baes P., Sühring S., Sajama J., Sotola E., Alonso-Pedano M., Bravo S., Godínez-Alvarez H., Ramawat K. (2009). Diversity and conservation in the Cactus family. Desert Plants. Biology and Biotechnology.

[15] Dubeux J.C.B., Dos Santos M.V.F., Da Cunha M.V., Dos Santos D.C., De Almeida Souza R.T., De Mello A.C.L., De Souza T.C. (2021). Cactus (*Opuntia* and *Nopalea*) nutritive value: A review. Anim. Feed Sci. and Tech..

[16] Ciriminna R., Chavarría-Hernández N., Rodríguez-Hernández A.I., Pagliaro M. (2019). Toward unfolding the bioeconomy of nopal (*Opuntia* spp.). Biofuel Bioprod. Bior..

[17] Le Houerou H.N. (2000). Utilization of fodder trees and shrubs in the arid and semiarid zones of West Asia and North Africa. Arid Soil Res. Rehabil..

[18] Silva M.A., Albuquerque T.G., Pereira P., Ramalho R., Vicente F., Oliveira M.B.P., Costa H.S. (2021). *Opuntia ficus-indica* (L.) Mill.: A multi-benefit potential to be exploited. Molecules.

[19] Bartomeus I., Vilà M. (2009). Breeding system and pollen limitation in two supergeneralist alien plants invading Mediterranean shrublands. Aust. J. Bot..

[20] Padrón B., Traveset A., Biedenweg T., Díaz D., Nogales M., Olesen J.M. (2009). Impact of alien plant invaders on pollination networks in two archipelagos. PLoS ONE.

[21] Instituto Nacional de Estadística y Geografía (INEGI) (2007). Características Principales del Cultivo del Nopal en el Distrito Federal Caso Milpa Alta.

[22] Rebman J.P., Pinkava D.J. (2001). *Opuntia* cacti of North America: An overview. Fla. Entomol..

[23] Reyes-Agüero J.A., Valiente-Banuet A. (2006). Reproductive biology of *Opuntia*: A review. J. Arid Environ..

[24] Inglese P., Mondragon C., Nefzaoui A., Saenz C., Taguchi M., Makkar H., Louhaichi M. (2018). Ecologia del Cultivo, Manejo y Usos del Nopal.

[25] Mandujano M.C., Carrillo-Ángeles I., Martínez Peralta C., Golubov J., Ramawat K. (2009). Reproductive biology of Cactaceae. Desert Plants. Biology and Biotechnology.

[26] Kearns C.A., Inouye D.W., Waser N.M. (1998). Endangered mutualisms: The conservation of plant-pollinator interactions. Annu. Rev. Ecol. Syst..

[27] Taki H., Kevan P.G. (2007). Does habitat loss affect the communities of plants and insects equally in plant–pollinator interactions? Preliminary findings. Biodivers. Conserv..

[28] Senapathi D., Biesmeijer J.C., Breeze T.D., Kleijn D., Potts S.G., Carvalheiro L.G. (2015). Pollinator conservation—The difference between managing for pollination services and preserving pollinator diversity. Curr. Opin. Insect Sci..

[29] Potts S.G., Biesmeijer J.C., Kremen C., Neumann P., Schweiger O., Kunin W.E. (2010). Global pollinator declines: Trends, impacts and drivers. Trends Ecol. Evol..

[30] Van der Kooi C.J., Vallejo-Marín M., Leonhardt S.D. (2021). Mutualisms and (A) symmetry in plant-pollinator interactions. Curr. Biol..

[31] Morales-Trejo J.J., Sandoval-Ruiz C.A., Fascinetto-Zago P., Cruzado-Lima A.L., Vázquez-Hernández C. (2014). Abundancia y diversidad de visitadores florales de *Opuntia pilifera* en Zapotitlán Salinas, Puebla. Entomol. Mex..

[32] Pimienta B.E., Del Castillo R.F., Nobel P.S. (2002). Reproductive biology. Cacti: Biology and Uses.

[33] Charbonneau D., Blonder B., Dornhaus A., Holme P., Saramaki J. (2013). Social insects: A model system for network dynamics. Temporal Networks, Understanding Complex Systems.

[34] Dáttilo W., Fagundes R., Gurka C.A., Silva M.S., Vieira M.C., Izzo T.J., Díaz-Castelazo C., Del-Claro K., Rico-Gray V. (2014). Individual-based ant-plant networks: Diurnal-nocturnal structure and species-area relationship. PLoS ONE.

[35] Ávila-Gómez E.S., Meléndez-Ramírez V., Castellanos I., Zuria I., Moreno C.E. (2019). Prickly pear crops as bee diversity reservoirs and the role of bees in *Opuntia* fruit production. Agric. Ecosyst. Environ..

[36] González A.B.D.J., García A.A., Olguín J.F.L., Rivera A., Martínez V.L. (2016). Entomofauna asociada al nopal verdura (*Opuntia ficusindica* Miller) en San Andrés Cholula, Puebla, México. Southwest Entomol..

[37] Del Castillo R., González-Espinosa M. (1988). Una interpretación evolutiva del polimorfismo sexual de *Opuntia robusta* (Cactaceae). Agrociencia.

[38] Mandujano M.D.C., Montaña C., Eguiarte L.E. (1996). Reproductive ecology and inbreeding depression in *Opuntia rastrera* (Cactaceae) in the Chihuahuan Desert: Why are sexually derived recruitments so rare?. Am. J. Bot..

[39] Mandujano M.C., Plasencia-López L., Aguilar-Morales G., Jiménez-Guzmán G., Galicia-Pérez A., Rojas-Aréchiga M., Martínez-Peralta C. (2014). Sistema sexual de *Opuntia tomentosa* Salm-Dyck (Cactaceae, Opuntioideae) en un pedregal de origen volcánico. Cactáceas Suculentas Mex..

[40] Muñoz-Urías A., Palomino-Hasbach G., Huerta-Martínez F.M., Pimienta-Barrios E., Ramírez-Hernández B.C. (2006). Reproductive isolation in fragmented wild populations of *Opuntia streptacantha*. J. Prof. Assoc. Cactus Dev..

[41] Piña H.H., Montaña C., Mandujano M. (2007). Fruit abortion in the Chihuahuan-Desert endemic cactus *Opuntia microdasys*. Plant Ecol..

[42] Sánchez-Echeverría K., Castellanos I., Mendoza-Cuenca L.F. (2016). Abejas visitantes florales de *Opuntia heliabravoana* en un gradiente de urbanización. Biológicas.

[43] Santa Anna-Aguayo A.I., Schaffner C.M., Golubov J., López-Portillo J., García-Franco J., Herrera-Meza G., Martínez A.J. (2017). Behavioral repertoires and interactions between *Apis mellifera* (Hymenoptera: Apidae) and the native bee *Lithurgus littoralis* (Hymenoptera: Megachilidae) in flowers of *Opuntia huajuapensis* (Cactaceae) in the Tehuacan desert. Fla. Entomol..

[44] Riojas-López M.E., Díaz-Herrera I.A., Fierros-López H.E., Mellink E. (2019). The effect of adjacent habitat on native bee assemblages in a perennial low-input agroecosystem in a semiarid anthropized landscape. Agric. Ecosyst. Environ..

[45] Gallegos-Vázquez C., Méndez-Gallegos S.D.J., Mondragón J.C. (2013). Producción Sustentable de Tuna en San Luis Potosí.

[46] Potgieter J., D’Aquino S., Inglese P., Mondragon C., Nefzaoui A., Saenz C., Taguchi M., Makkar H., Louhaichi M. (2018). Fruit production and post-harvest management. Ecologia del Cultivo, Manejo y Usos del Nopal.

[47] Riojas-López M., Mellink E. (2005). Potential for biological conservation in man-modified semiarid habitats in northeastern Jalisco, Mexico. Biodivers. Conserv..

[48] Bembower W. (1911). Pollination notes from the Cedar Point region. Ohio Nat..

[49] Cockerell T.D.A. (1922). Two new subgenera of north American bees. Am. Mus. Novit.

[50] Grant V., Grant K.A. (1979). Pollination of *Opunta basilaris* and *O. littoralis*. Plant Syst. Evol..

[51] Grant V., Grant K.A., Hurd P.D. (1979). Pollination of *Opuntia lindheimeri* and related species. Plant Syst. Evol..

[52] Fachardo A.L.S., Sigrist M.R. (2020). Pre-zygotic reproductive isolation between two synchronopatric *Opuntia* (Cactaceae) species in the Brazilian Chaco. Plant Biol..

[53] Pretto F., Celesti-Grapow L., Carli E., Blasi C. (2010). Influence of past land use and current human disturbance on non-native plant species on small Italian islands. Plant Ecol..

[54] Lo Verde G., La Mantia T. (2011). The role of native flower visitors in pollinating *Opuntia ficus-indica* (L.) Mill., naturalized in Sicily. Acta Oecologica.

[55] Bartomeus I., Vilà M., Santamaría L. (2008). Contrasting effects of invasive plants in plant–pollinator networks. Oecologia.

[56] Jauker F., Speckmann M., Wolters V. (2016). Intra-specific body size determines pollination effectiveness. Basic Appl. Ecol..

[57] Osborn M.M., Kevan P.G., Lane M.A. (1988). Pollination biology of *Opuntia polyacantha* and *Opuntia phaeacantha* (Cactaceae) in southern Colorado. Plant Syst. Evol..

[58] Cota-Sánchez J.H., Almeida O.J.G., Falconer D.J., Choi H.J., Bevan J. (2013). Intriguing thigmonastic (sensitive) stamens in the plains prickly pear *Opuntia polyacantha* (Cactaceae). Flora.

[59] Schlindwein C., Wittmann D. (1997). Stamen movements in flowers of *Opuntia* (Cactaceae) favour oligolectic pollinators. Plant Syst. Evol..

[60] Lenzi M., Orth A.I. (2011). Floral visitors of the *Opuntia monacantha* (Cactaceae) in sandbank of the Florianópolis, SC, Brazil. Acta Biológica Paranaense.

[61] Gómez J.M. (2000). Effectiveness of ants as pollinators of *Lobularia maritima*: Effects on main sequential fitness components of the host plant. Oecologia.

[62] Maubecin C.C., Boero L., Sérsic A.N. (2020). Specialisation in pollen collection, pollination interactions and phenotypic variation of the oil-collecting bee *Chalepogenus cocuccii*. Apidologie.

[63] Arroyo-Pérez E., Jiménez-Sierra C.L., Zavala Hurtado J.A., Flores J. (2021). Shared pollinators and sequential flowering phenologies in two sympatric cactus species. Plant Ecol. Evo..

[64] Samra S., Samocha Y., Eisikowitch D., Vaknin Y. (2014). Can ants equal honeybees as effective pollinators of the energy crop *Jatropha curcas* L. under Mediterranean conditions?. GCB Bioenergy.

[65] Pickett C.H., Clark W.D. (1979). The function of extrafloral nectaries in *Opuntia acanthocarpa* (Cactaceae). Am. J. Bot..

[66] LeVan K.E., Hung K.L.J., McCann K.R., Ludka J.T., Holway D.A. (2014). Floral visitation by the Argentine ant reduces pollinator visitation and seed set in the coast barrel cactus, *Ferocactus viridescens*. Oecologia.

[67] Mauseth J.D., Rebmann J.P., Machado S.R. (2016). Extrafloral nectaries in cacti. Cactus Succul. J..

[68] Wagner D., Kay A. (2002). Do extrafloral nectaries distract ants from visiting flowers? An experimental test of an overlooked hypothesis. Evol. Ecol. Res..

[69] Komamura R., Koyama K., Yamauchi T., Konno Y., Gu L. (2021). Pollination contribution differs among insects visiting *Cardiocrinum cordatum* flowers. Forests.

[70] Beattie A.J., Turnbull C., Knox R.B., Williams E.G. (1984). Ant inhibition of pollen function—A possible reason why ant pollination is rare. Am. J. Bot..

[71] Rostás M., Bollmann F., Saville D., Riedel M. (2018). Ants contribute to pollination but not to reproduction in a rare calcareous grassland forb. PeerJ.

[72] Rostás M., Tautz J., Dubinsky Z., Seckbach J. (2010). Ants as pollinators of plants and the role of floral scents. All Flesh is Grass. Cellular Origin, Life in Extreme Habitats and Astrobiology.

[73] Gallai N., Salles J.M., Settele J., Vaissière B.E. (2009). Economic valuation of the vulnerability of world agriculture confronted with pollinator decline. Ecol. Econ..

[74] Rzedowski J. (1968). Las principales zonas áridas de México y su vegetación. Bios Revista del Seminario de Estudios Biológicos.

[75] Soberon J., Golubov J., Sarukhán J. (2001). The importance of *Opuntia* in Mexico and routes of invasion and impact of *Cactoblastis cactorum* (Lepidoptera: Pyralidae). Fla. Entomol..

[76] Vanegas-Rico J.M., Lomeli–Flores J.R., Rodríguez–Leyva E., Mora–Aguilera G., Valdez J.M. (2010). Natural enemies of *Dactylopius opuntiae* (Cockerell) on *Opuntia ficus-indica* (L.) Miller in Central México. Acta Zoológica Mex..

[77] McCravy K.W. (2018). A review of sampling and monitoring methods for beneficial arthropods in agroecosystems. Insects.

[78] Montgomery G.A., Belitz M.W., Guralnick R.P., Tingley M.W. (2021). Standards and best practices for monitoring and benchmarking insects. Front. Ecol. Evol..

[79] Vigueras A.L., Portillo L. (2001). Uses of *Opuntia* species and the potential impact of *Cactoblastis cactorum* (Lepidoptera: Pyralidae) in Mexico. Fla. Entomol..

[80] Griffith M.P. (2004). The origins of an important cactus crop, *Opuntia ficus-indica* (Cactaceae): New molecular evidence. Am. J. Bot..

[81] Barbera G., Carimi F., Inglese P. (1992). Past and present role of the indian-fig prickly-pear (*Opuntia ficus-indica* (L) Miller, Cactaceae) in the agriculture of Sicily. Econ. Bot..

[82] Stang M., Klinkhamer P.G., Van Der Meijden E. (2006). Size constraints and flower abundance determine the number of interactions in a plant–flower visitor web. Oikos.

[83] Lopezaraiza-Mikel M.E., Hayes R.B., Whalley M.R., Memmott J. (2007). The impact of an alien plant on a native plant–pollinator network: An experimental approach. Ecol. Lett..

[84] Ness J.H. (2020). Hot spots and hot moments for on-plant foraging by ants within the flora of warm North American Deserts. Am. Midl. Nat..

[85] Vithanage V. (1990). The role of the European honeybee (*Apis mellifera* L.) in avocado pollination. J. Hortic. Sci..

[86] Rizzardo R.A., Milfont M.O., Silva E., Freitas B.M. (2012). *Apis mellifera* pollination improves agronomic productivity of anemophilous castor bean (*Ricinus communis*). Anais da Academia Brasileira de Ciências.

[87] Moritz R.F., Härtel S., Neumann P. (2005). Global invasions of the western honeybee (*Apis mellifera*) and the consequences for biodiversity. Ecoscience.

[88] De la Rúa P., Paxton R.J., Moritz R.F.A., Roberts S., Allen D.J., Pinto M.A., Cauia E., Fontana P., Kryger P., Bouga M. *Apis mellifera*. The IUCN Red List of Threatened Species 2014, e.T42463639A42463665. https://ec.europa.eu/environment/nature/conservation/species/redlist/downloads/European_bees.pdf.

[89] Paudel Y.P., Mackereth R., Hanley R., Qin W. (2015). Honeybees (*Apis mellifera* L.) and pollination issues: Current status, impacts, and potential drivers of decline. J. Agric. Sci..

[90] Grixti J.C., Wong L.T., Cameron S.A., Favret C. (2009). Decline of bumble bees (*Bombus*) in the North American Midwest. Biol. Conserv..

[91] Jacobson M.M., Tucker E.M., Mathiasson M.E., Rehan S.M. (2018). Decline of bumble bees in northeastern North America, with special focus on *Bombus terricola*. Biol. Conserv..

[92] Hatfield R., Jepsen S., Thorp R., Richardson L., Colla S., Foltz Jordan S. *Bombus pensylvanicus*. The IUCN Red List of Threatened Species 2015, e.T21215172A21215281.

[93] SEMARNAT (2010). Norma Oficial Mexicana NOM-059-SEMARNAT-2010, Protección Ambiental-Especies Nativas de México de Flora y Fauna Silvestres-Categorías de Riesgo y Especificaciones para su Inclusión, Exclusión o Cambio-Lista de Especies en Riesgo.

[94] Hernández H.M., Gómez-Hinostrosa C., Goettsch B.K. *Opuntia chaffeyi*. The IUCN Red List of Threatened Species 2013, e.T41222A2952609.

[95] Hernández H.M., Gómez-Hinostrosa C., Goettsch B.K., Sotomayor M. *Opuntia megarrhiza*. The IUCN Red List of Threatened Species 2013, e.T41219A2952324.

[96] Majure L., Griffith P. *Opuntia abjecta*. The IUCN Red List of Threatened Species 2013, e.T199640A2608155.

[97] Nassar J., Majure L., Griffith P. *Opuntia schumannii*. The IUCN Red List of Threatened Species 2017, e.T152123A121580174.

[98] Majure L., Griffith P., Gann G.D. *Opuntia triacantha*. The IUCN Red List of Threatened Species 2017, e.T152237A121584692.

[99] Nassar J., Majure L. *Opuntia curassavica* (Amended Version of 2013 Assessment). The IUCN Red List of Threatened Species 2017, e.T152801A121610952.

[100] Negrón-Ortiz V. (1998). Reproductive biology of a rare cactus, *Opuntia spinosissima* (Cactaceae), in the Florida Keys: Why is seed set very low?. Sex. Plant Reprod..

[101] Areces-Mallea A.E. (2001). A new opuntioid cactus from the Cayman Islands, B.W.I., with a discussion and key to the genus *Consolea* Lemaire. Brittonia.

[102] Locatelli E., Machado I.C.S. (1999). Comparative study of the floral biology in two ornithophilous species of Cactaceae: *Melocactus zehntneri* and *Opuntia palmadora*. Bradleya.

[103] Akcelrad Lerner L. (2021). Comisión Nacional Para el Conocimiento y Uso de la Biodiversidad C. Especies Silvestres de Nopales Mexicanos. Version 1.9. Comisión Nacional Para el Conocimiento y Uso de la Biodiversidad. Occurrence Dataset. GBIF.org.

[104] Jordano P., Vázquez D., Bascompte J., Medel R., Aizen M.A., Zamora R. (2009). Redes complejas de interacciones mutualistas planta-animal. Ecología y Evolución de Interacciones Planta-Animal.

[105] Dáttilo W., Guimarães P.R., Izzo T.J. (2013). Spatial structure of ant–plant mutualistic networks. Oikos.

[106] Agüero J.I., Galati B.G., Torretta J.P. (2018). Structure and ultrastructure of floral nectaries of two *Opuntia* species (Cactaceae) in relation to their floral visitors. Plant Syst. Evol..

[107] Díaz L., Cocucci A.A. (2003). Functional gynodioecy in *Opuntia quimilo* (Cactaceae), a tree cactus pollinated by bees and hummingbirds. Plant Biol..

[108] Janeba Z. (2009). Insect flower visitors and pollinators of cacti from the southwest USA. Bradleya.

[109] Ribbens E., Anderson B.A., Fant J.B. (2011). *Opuntia fragilis* (Nuttall) Haworth in Illinois: Pad dynamics and sexual reproduction. Haseltonia.

[110] Mandujano M.C. (2013). Reproductive ecology of *Opuntia macrocentra* (Cactaceae) in the northern Chihuahuan Desert. Am. Midl. Nat..

[111] De Castro Verçoza F. (2019). Comportamento de *Xylocopa ordinaria* Smith (Hymenoptera, apidae) na polinização de espécies em duas comunidades vegetais de restinga do município do Rio de Janeiro, Brasil. Revista Dissertar.

[112] Spears E.E. (1987). Island and mainland pollination ecology of *Centrosema virginianum* and *Opuntia stricta*. J. Ecol..

