# Supplementary material for: A Systematic Review on Opuntia (Cactaceae; Opuntioideae) Flower-Visiting Insects in the World with Emphasis on Mexico: Implications for Biodiversity Conservation
Source: Plants (Basel). 2022 Jan 4;11(1):131. doi: 10.3390/plants11010131 (PMC8747471; doi:10.3390/plants11010131)
Supplement: Supplementary file 1 [file plants-11-00131-s001.zip › Supplementary Materials Table S1.pdf]

Table S1. Checklist of the 29 *Opuntia* species with their associated insect flower-visiting species. This database was extracted from the 29 retrieved articles published from 1911 to 2020 worldwide. The references cited herein can be found in the References list in the main manuscript. Note that insect species are listed according to the Order and Family they belong to.

Opuntia species	Insect species	Country	References
***Opuntia anacantha* Speg.**	**Coleoptera**<br>**Buprestidae:** *Tetragonoschema* sp. Thomson, 1857<br>**Nitidulidae:** *Carpophilus* sp. Stephens, 1829<br>**Hymenoptera**<br>**Apidae:** *Alepidosceles filitarsis* Vachal, 1904; *Ceratina* sp. Latreille, 1802; *Diadasia* sp. (2) Patton, 1879; *Ptilothrix scalaris* Holmberg, 1903; *Ptilothrix* sp. Smith, 1853; *Ptilothrix tricolor* Friese, 1906; *Tetrapedia* sp. Klug, 1810<br>**Halictidae:** *Augochlora amphitrite* Schrottky, 1909; *Lasioglossum* sp. Curtis, 1833<br>**Megachilidae:** *Lithurgus rufiventris* Friese, 1908; *Lithurgus* sp. Berthold, 1827; *Megachile* sp. Latreille, 1802	Argentin a	[106,107]
***Opuntia basilaris* Engelm & Bigelow**	**Coleoptera**<br>**Melyridae:** *Trichochrous umbratus* LeC.<br>**Nitidulidae:** *Nitops pallipennis* Say, 1823<br>**Hymenoptera**<br>**Apidae:** *Anthophora fulvicauda* Timberlake, 1940; *Diadasia australis californica* Timberlake, 1940; *Diadasia rinconis* Cockerell, 1897<br>**Halictidae:** *Agapostemon angelicus* or *texanus*; *Lasioglossum* sp. Curtis, 1833	U.S.A.	[50,108]
***Opuntia dillenii* (Ker Gawl.) Haw.**	**Hymenoptera**<br>**Apidae:** *Apis mellifera* Linnaeus, 1758; *Bombus terrestris* Linnaeus, 1758<br>**Halictidae:** *Lasioglossum viride* Brullé, 1839	Spain	[20]

Opuntia species	Insect species	Country	References
***Opuntia elata* Link & Otto ex Salm-Dyck**	**Coleoptera**<br>**Buprestidae:** *Tetragonoschema* sp. Thomson, 1857<br>**Chrysomelidae:** *Chrysomelidae* sp. **(2)** Latreille, 1802<br>**Nitidulidae:** *Carpophilus* sp. Stephens, 1829<br>**Hymenoptera**<br>**Andrenidae:** *Arhysosage flava* Moure 1958; *Arhysosage* sp. Brèthes, 1922<br>**Apidae:** *Apidae* sp. Latreille, 1802; *Bombus morio* Swederus, 1787; *Diadasia patagonica* Brèthes, 1910; *Diadasia* sp. Patton, 1879; *Plebeia molesta* Puls, 1869; *Ptilothrix tricolor* Friese, 1906<br>**Halictidae:** *Augochlora* sp. Smith, 1853; *Augochloropsis* sp. Cockerell, 1897<br>**Megachilidae:** *Lithurgopsis cf. rufiventris* Friese, 1908; *Lithurgus rufiventris* Friese, 1908; *Lithurgus* sp. Berthold, 1827; *Megachile* sp. Latreille, 1802<br>**Vespidae:** *Polybia ignobilis* Haliday, 1836<br>**Lepidoptera**<br>**Lepidoptera:** *Lepidoptera* sp. Linnaeus, 1758<br>**Pieridae:** *Phoebis* sp. Hübner, 1819; *Pieridae* sp. Duponchel, 1835	Argentin a, Brazil	[52,106, 107]
***Opuntia engelmannii* Salm-Dyck ex Engelm.**	**Hymenoptera**<br>**Apidae:** *Diadasia rinconis* Cockerell, 1897	U.S.A.	[108]
***Opuntia ficus-indica* (L.) Mill., 1768**	**Coleoptera**<br>**Cerambycidae:** *Phytoecia* sp. Dejean, 1835; *Stenopterus ater* Linnaeus, 1767; *Stictoleptura cordigera* Fuessly, 1775<br>**Chrysomelidae:** *Chrysomelidae* sp. Latreille, 1802; *Lachnaia paradoxa* Olivier, 1808; *Tituboea biguttata* Olivier, 1791<br>**Cleridae:** *Trichodes alvearius* Fabricius, 1792<br>**Coccinellidae:** *Coccinella septempunctata* Linnaeus, 1758; *Coccinellidae* sp. Latreille, 1807<br>**Curculionidae:** *Curculionidae* sp. Latreille, 1802<br>**Elateridae:** *Cebrio melanocephalus* Leach, 1824<br>**Lampyridae:** *Lampyris* sp. Geoffroy, 1762<br>**Melyridae:** *Divales bipustulatus* Fabricius, 1781; *Melyridae* sp. Leach, 1815	Argentin a, Italy, Mexico, U.S.A	[47,54,107]

Opuntia species	Insect species	Country	References
	**Mycteridae:** *Mycterus* sp. Clairville, 1798<br>**Oedemeridae:** *Oedemera barbara* Fabricius, 1792; *Oedemera flavipes* Fabricius, 1792; *Oedemera nobilis* Scopoli, 1763; *Oedemera simplex* Linnaeus, 1767<br>**Scarabaeidae:** *Aethiessa floralis* Fabricius, 1787; *Cetonia aurata* Linnaeus, 1758; *Oxythyrea funesta* Poda, 1761; *Paratriodonta cinctipennis* Lucas, 1846; *Protaetia cuprea* Fabricius, 1775; *Tropinota hirta* Poda, 1761<br>**Ptinidae:** *Ptinidae* sp. Latreille, 1802<br>**Tenebrionidae:** *Omophlus lepturoides* Fabricius, 1787; *Omophlus* sp. Dejean, 1834<br>**Dermaptera**<br>**Forficulidae:** *Forficula auricularia* Linnaeus, 1758; *Forficula decipiens* Géné, 1832<br>**Labiduridae:** *Labidura riparia* Pallas, 1773<br>**Diptera**<br>**Diptera:** *Diptera* sp. Linnaeus, 1758<br>**Hemiptera**<br>**Anthocoridae:** *Orius laevigatus* Fieber, 1860; *Orius niger* Wolff, 1811<br>**Aphrophoridae:** *Philaenus spumarius* Linnaeus, 1758<br>**Cicadellidae:** *Cicadellidae* sp. Latreille, 1802; *Eupteryx zelleri* Kirschbaum, 1868<br>**Issidae:** *Issidae* sp. Spinola, 1839<br>**Miridae:** *Taylorilygus apicalis* Fieber, 1861<br>**Pentatomidae:** *Graphosoma semipunctatum* Fabricius, 1775<br>**Hymenoptera**<br>**Andrenidae:** *Arhysosage* sp. Brèthes, 1922; *Macrotera azteca* Timberlake, 1954; *Macrotera bicolor* Smith, 1853<br>**Apidae:** *Anthophora montana* Cresson, 1869; *Apis mellifera* Linnaeus, 1758; *Bombus hortorum* Linnaeus, 1761; *Bombus morio* Swederus, 1787; *Bombus pascuorum* Scopoli, 1763; *Bombus terrestris* Linnaeus, 1758; *Ceratina* aff. *nanula* Cockerell, 1897; *Ceratina* aff. *neomexicana* Cockerell, 1901; *Ceratina cucurbitina* Rossi, 1972; *Ceratina* sp. Latreille, 1802; *Diadasia patagonica* Brèthes, 1910; *Diadasia rinconis* Cockerell, 1897; *Melipona* sp. Illiger, 1806; *Melissodes gilensis* Cockerell, 1896; *Melissodes tristis* Cockerell, 1894; *Ptilothrix tricolor* Friese, 1906; *Xylocopa* sp. Latreille, 1802; *Xylocopa violacea* Linnaeus, 1758<br>**Chalcidoidea:** *Chalcidoidea* sp.		

Opuntia species	Insect species	Country	References
	**Formicidae:** *Formicidae* sp. Latreille, 1809<br>**Halictidae:** *Agapostemon texanus* Cresson, 1872; *Agapostemon tyleri* Cockerell, 1917; *Augochloropsis metallica* Fabricius, 1793; *Augochloropsis* sp. Cockerell, 1897; *Halictus fulvipes* Klug, 1817; *Halictus ligatus* Say, 1837; *Halictus scabiosae* Rossi, 1790; *Lasioglossum opacum* Moure, 1940; *Lasioglossum smeathmanellum* Kirby, 1802; *Lasioglossum* sp. **(8)** Curtis, 1833<br>**Ichneumonidae:** *Ichneumonidae* sp. Latreille, 1802<br>**Megachilidae:** *Ashmeadiella cactorum* Cockerell, 1897; *Lithurgus littoralis* Cockerell, 1917; *Lithurgus* sp. Berthold, 1827; *Megachile* sp. Latreille, 1802; *Rhodanthidium septemdentatum* Latreille, 1809; *Rhodanthidium sticticum* Fabricius, 1787<br>**Sphecidae:** *Sphecidae* sp. Latreille, 1802<br>**Vespidae:** *Polistes gallicus* Linnaeus, 1767; *Polybia ignobilis* Haliday, 1836<br>**Lepidoptera**<br>**Nymphalidae:** *Vanessa atalanta* Linnaeus, 1758<br>**Pieridae:** *Pieris rapae* Linnaeus, 1758<br>**Neuroptera**<br>**Chrysopidae:** *Chrysopidae* sp. Schneider, 1851<br>**Orthoptera**<br>**Tettigoniidae:** *Tettigonia viridissima* Linnaeus, 1758<br>**Thysanoptera**<br>*Thysanoptera* sp. Haliday, 1836		

Opuntia species	Insect species	Country	References
***Opuntia fragilis* (Nutt.) Haw.**	**Coleoptera**<br>**Scarabaeidae:** *Scarabaeidae* sp. Latreille, 1802<br>**Diptera**<br>*Diptera* sp. Linnaeus, 1758<br>**Hymenoptera**<br>**Apidae:** *Bombus* sp. Latreille, 1802<br>**Halictidae:** *Halictidae* sp. Thomson, 1869<br>**Megachilidae:** *Megachilidae* sp. Latreille, 1802<br>**Lepidoptera**<br>**Lepidoptera:** *Lepidoptera* sp. Linnaeus, 1758<br>**Orthoptera**<br>**Acrididae:** *Acrididae* sp. MacLeay, 1821	U.S.A.	[109]
***Opuntia huajuapensis* Bravo, 1954**	**Hymenoptera**<br>**Apidae:** *Apis mellifera* Linnaeus, 1758; *Diadasia* sp. Patton, 1879<br>**Megachilidae:** *Lithurgus littoralis* Cockerell, 1917	Mexico	[43]
***Opuntia humifusa* Raf.**	**Coleoptera**<br>**Curculionidae:** *Odontocorynus umbellae* Fabricius, 1802<br>**Scarabaeidae:** *Strigoderma arboricola* Fabricius, 1792; *Trichiotinus piger* Fabricius, 1775<br>**Hymenoptera**<br>**Apidae:** *Bombus pensylvanicus* De Geer, 1773; *Ceratina dupla* Say, 1837<br>**Crabronidae:** *Microbembex monodonta* Say, 1824<br>**Halictidae:** *Agapostemon radiatus* Forster, 1771<br>**Vespidae:** *Vespa borealis* Lewis, 1897	U.S.A.	[48]

Opuntia species	Insect species	Country	References
***Opuntia lindheimeri* Engelm.**	**Coleoptera**<br>**Buprestidae:** *Acmaeodera neglecta* Fall, 1899; *Acmaeodera ornatoides* Barr, 1972<br>**Chrysomelidae:** *Brachypnoea puncticollis* Say, 1824; *Diabrotica undecimpunctata* howardi Barber, 1947<br>**Malachiidae:** *Hypebaeus* sp. Kiesenwetter, 1863<br>**Melyridae:** *Tanaops* sp. LeConte, 1859; *Trichochrous* sp. Motschulsky, 1859<br>**Nitidulidae:** *Carpophilus floralis* Erichson, 1843<br>**Scarabaeidae:** *Euphoria kernii* Haldeman, 1852<br>**Hymenoptera**<br>**Andrenidae:** *Macrotera texana* Cresson, 1878<br>**Apidae:** *Apis mellifera* Linnaeus, 1758; *Ceratina dupla* Say, 1837; *Diadasia rinconis* Cockerell, 1897; *Melissodes tristis* Cockerell, 1894; *Xylocopa virginica texana* Cresson, 1872<br>**Halictidae:** *Agapostemon texanus* Cresson, 1872; *Lasioglossum pilosum* Smith, 1853<br>**Megachilidae:** *Lithurgus gibbosus* Smith, 1853; *Megachile amica* Cresson, 1872	U.S.A.	[51]
***Opuntia littoralis* Engelm.**	**Coleoptera**<br>**Nitidulidae:** *Nitops pallipennis* Say, 1823<br>**Hymenoptera**<br>**Apidae:** *Diadasia australis californica* Timberlake, 1940	U.S.A.	[50]
***Opuntia macrocentra* Engelm.**	**Hymenoptera**<br>**Apidae:** *Apidae* sp. Latreille, 1802; *Diadasia* aff. *rinconis* Cockerell, 1897; *Melissodes* sp. Latreille, 1829	U.S.A.	[110]

Opuntia species	Insect species	Country	References
***Opuntia macrorhiza* Engelm.**	**Coleoptera**<br>**Cerambycidae:** *Moneilema* sp.<br>**Meloidae:** *Epicauta* sp. Dejean, 1834<br>**Hymenoptera**<br>**Andrenidae:** *Macrotera opuntiae* Cockerell, 1922<br>**Apidae:** *Anthophorula cornigera* Cockerell, 1922; *Exomalopsis solani* Cockerell, 1896<br>**Colletidae:** *Colletes* sp. Latreille, 1802<br>**Halictidae:** *Agapostemon texanus* Cresson, 1872<br>**Megachilidae:** *Lithurgus apicalis* Cresson, 1875	U.S.A.	[49]
***Opuntia maxima* Mill.**	**Coleoptera**<br>**Oedemeridae:** *Oedemera* sp. Olivier, 1789<br>**Scarabaeidae:** *Oxythyrea funesta* Poda, 1761<br>**Staphylinidae:** *Stenopleurus* sp. Kistner 1997<br>**Hymenoptera**<br>**Apidae:** *Apis mellifera* Linnaeus, 1758; *Bombus* terrestris Linnaeus, 1758; Xylocopa violacea Linnaeus, 1758<br>**Halictidae:** *Halictus scabiosae* Rossi, 1790<br>**Megachilidae:** *Rhodanthidium septemdentatum* Latreille, 1809<br>**Lepidoptera**<br>**Pieridae:** *Gonepteryx cleopatra* Linnaeus, 1767	Spain	[20]
***Opuntia microdasys* (Lehm.) Pfeiff.**	**Coleoptera**<br>**Buprestidae:** *Acmaeodera* sp. Eschscholtz, 1829<br>**Nitidulidae:** *Nitidulidae* sp. **(2)** Latreille, 1802<br>**Hymenoptera**<br>**Andrenidae:** *Perdita* sp. Smith, 1853<br>**Apidae:** *Diadasia* sp. Patton, 1879<br>**Megachilidae:** *Ashmeadiella* sp. Cockerell, 1897; *Megachile* sp. Latreille, 1802	Mexico	[41]

Opuntia species	Insect species	Country	References
***Opuntia monacantha* (Willd.) Haw.**	**Coleoptera**<br>**Nitidulidae:** *Camptodes* sp. Erichson, 1843<br>**Scarabaeidae:** *Cyclocephala* sp. Dejean, 1821<br>**Hymenoptera**<br>**Andrenidae:** *Arhysosage cactorum* Moure, 1999; *Callonychium petuniae* Cure and Wittman, 1990; *Panurgillus* sp. Moure, 1998<br>**Apidae:** *Alloscirtetica gilva* Holmberg, 1884; *Ancyloscelis romeroi* Holmberg, 1903; *Apis mellifera* Linnaeus, 1758; *Bombus morio* Swederus, 1787; *Bombus pauloensis* Friese, 1912; *Ceratina asuncionis* Strand, 1910; *Ceratina hyemalis* Moure, 1950; *Ceratina ischiocentra* Moure; *Ceratina morrensis* Strand, 1910; *Ceratina rupestris* Holmberg, 1884; *Chalepogenus goeldianus* Friese, 1899; *MelissoptiIa bonaerensis* Holmberg; *Mourella caerulea* Friese, 1900; *Plebeia emerina* Friese, 1900; *Plebeia wittmanni* Moure & Camargo, 1989; *Ptilothrix fructifera* Holmberg, *1903; Tetrapedia rugulosa* Friese, 1899; *Trigona spinipes* Fabricius, 1793; *Xylocopa augusti* Lepeletier, 1841; *Xylocopa brasilianorum* Linnaeus, 1767; *Xylocopa frontalis* Olivier, 1789; *Xylocopa ordinaria* Smith, 1874<br>**Colletidae:** *Cephalocolletes rugata* Urban; *Leioproctus isabelae* Urban, 1995; *Sarocolletes* sp. Michener, 1989<br>**Halictidae:** *Augochlora amphitrite* Schrottky, 1909; *Augochlora daphnis* Smith, 1853; *Augochlora semiramis* Schrottky, 1910; *Augochlora* sp. (3) Smith, 1853; *Augochlorella michaelis* Vachal, 1911; *Augochloropsis cupreola* Cockerell, 1900; *Augochloropsis euterpe* Holmberg, 1886; *Augochloropsis* sp. Cockerell, 1897; *Dialictus* sp. **(3)** Robertson, 1902; *Lasioglossum opacum* Moure, 1940; *Thectochlora alaris* Vachal, 1904<br>**Megachilidae:** *Lithurgus rufiventris* Friese, 1908; *Megachile gomphrenoides* Vachal, 1908; *Megachile* sp. **(3)** Latreille, 1802; *Microthurge pygmaeus* Friese, 1908	Brazil	[59,60,111]

Opuntia species	Insect species	Country	References
***Opuntia phaeacantha* Engelm.**	**Hymenoptera**<br>**Apidae:** *Diadasia rinconis* Cockerell, 1897; *Melissodes* sp. Latreille, 1829<br>**Halictidae:** *Agapostemon texanus* Cresson, 1872; *Augochlorella aurata* Smith<br>**Megachilidae:** *Ashmeadiella opuntiae* Cockerell, 1897; *Lithurgus apicalis* Cresson, 1875; *Megachile casadae* Cockerell, 1898	U.S.A.	[57]
***Opuntia pilifera* Weber, 1898**	**Coleoptera**<br>*Coleoptera* sp. **(10)** Linnaeus, 1758<br>**Hemiptera**<br>*Hemiptera* sp. **(15)** Linnaeus, 1758<br>**Hymenoptera**<br>*Hymenoptera* sp. **(24)** Linnaeus, 1758<br>**Lepidoptera**<br>*Lepidoptera* sp. Linnaeus, 1758<br>**Neuroptera**<br>*Neuroptera* sp. Linnaeus, 1758	Mexico	[32]

Opuntia species	Insect species	Country	References
***Opuntia polyacantha* Haw.**	**Coleoptera**<br>**Cerambycidae:** *Batyle aff. suturalis* Say, 1823<br>**Mordellidae:** *Mordellidae* sp. Latreille, 1802<br>**Nitidulidae:** *Nitops aff. pallipennis* Say, 1823; *Nitops pallipennis* Say, 1823<br>**Diptera**<br>**Bombyliidae:** *Systoechus vulgaris* Loew, 1863<br>**Hymenoptera**<br>**Apidae:** *Apis* sp. Linnaeus, 1758; *Bombus pensylvanicus* De Geer, 1773; *Bombus* sp. Latreille, 1802; *Diadasia australis* Cresson, 1879; *Diadasia diminuta* Cresson, 1879<br>**Formicidae:** *Formicidae* sp. Latreille, 1809<br>**Halictidae:** *Agapostemon coloradinus* Vachal, 1903; *Agapostemon* sp. Guérin-Méneville, 1844; *Agapostemon texanus* Cresson, 1872; *Halictus confusus* Smith, 1853; *Lasioglossum* sp. Curtis, 1833<br>**Megachilidae:** *Lithurgus apicalis* Cresson, 1875; *Megachile casadae* Cockerell, 1898; *Megachile concinna* Smith, 1879; *Megachile dentitarsus* Sladen, 1919; *Megachile montivaga* Cresson, 1878<br>**Lepidoptera**<br>*Lepidoptera* sp. **(2)** Linnaeus, 1758<br>**Orthoptera**<br>**Acrididae:** *Cannula* sp. Bolívar, 1906	U.S.A., Canada	[57,58,108]
***Opuntia quimilo* K. Schum.**	**Hymenoptera**<br>**Apidae:** *Bombus morio* Swederus, 1787; *Diadasia patagonica* Brèthes, 1910; *Ptilothrix tricolor* Friese, 1906; *Xylocopa* sp. Latreille, 1802<br>**Megachilidae:** *Lithurgus* sp. Berthold, 1827; *Megachile* sp. Latreille, 1802<br>**Vespidae:** *Polybia ignobilis* Haliday, 1836<br>**Lepidoptera**<br>**Pieridae:** *Pieridae* sp. Duponchel, 1835	Argentin a	[107]

Opuntia species	Insect species	Country	References
***Opuntia rastrera* F.A.C. Weber**	**Coleoptera**<br>*Nitidulidae* sp. **(2)** Latreille, 1802<br>**Hymenoptera**<br>**Andrenidae:** *Perdita* sp. Smith, 1853<br>**Apidae:** *Diadasia* sp. Patton, 1879; *Melissodes* sp. Latreille, 1829<br>**Formicidae:** *Formicidae* sp. **(2)** Latreille, 1809<br>**Halictidae:** *Lasioglossum* sp. Curtis, 1833<br>**Megachilidae:** *Ashmeadiella* sp. Cockerell, 1897; *Lithurgus* sp. Berthold, 1827; *Megachile* sp. Latreille, 1802<br>**Vespidae:** *Vespidae* sp. Latreille, 1802	Mexico	[38]
***Opuntia retrorsa* Speg. 1905**	**Hymenoptera**<br>**Andrenidae:** *Arhysosage biguttulata* Ramos, 2013<br>**Apidae:** *Ceratina* sp. **(2)** Latreille, 1802<br>**Megachilidae:** *Lithurgopsis cf. rufiventris* Friese, 1908; *Megachile barbiellinii* Moure, 1944	Brazil	[52]

Opuntia species	Insect species	Country	References
***Opuntia robusta* J.C. Wendl.**	**Coleoptera**<br>**Buprestidae:** *Acmaeodera picta* Waterhouse, 1882; *Acmaeodera scalaris* Mannerheim, 1837<br>**Melyridae:** *Trichochrous* sp. Motschulsky, 1859<br>**Nitidulidae:** *Camptodes* sp. Erichson, 1843; *Carpophilus* sp. Stephens, 1829<br>**Scarabaeidae:** *Euphoria* sp. Burmeister, 1842<br>**Hymenoptera**<br>**Andrenidae:** *Macrotera azteca* Timberlake, 1954; *Macrotera bicolor* Smith, 1853<br>**Apidae:** *Bombus sonorus* Say, 1837; *Diadasia rinconis* Cockerell, 1897; *Melissodes tristis* Cockerell, 1894<br>**Halictidae:** *Agapostemon* sp. Guérin-Méneville, 1844; *Agapostemon texanus* Cresson, 1872; *Augochlorella* sp. Sandhouse, 1937; *Halictidae* sp. Thomson, 1869; *Lasioglossum* sp. Curtis, 1833<br>**Megachilidae:** *Ashmeadiella* sp. Cockerell, 1897; *Lithurgus littoralis* Cockerell, 1917	Mexico	[37]
***Opuntia spinulifera* Salm-Dyck**	**Hymenoptera**<br>**Andrenidae:** *Macrotera bicolor* Smith, 1853; *Macrotera sinaloana* Timberlake, 1958<br>**Apidae:** *Apis mellifera* Linnaeus, 1758; *Bombus* sp. Latreille, 1802; *Ceratina* sp. Latreille, 1802; *Diadasia diminuta* Cresson, 1879; *Diadasia* sp. Patton, 1879<br>**Halictidae:** *Agapostemon leunculus* Vachal, 1903; *Agapostemon* sp. Guérin-Méneville, 1844; *Lasioglossum* sp. **(6)** Curtis, 1833<br>**Megachilidae:** *Lithurgus littoralis* Cockerell, 1917	Mexico	[42]

Opuntia species	Insect species	Country	References
***Opuntia streptacantha* Lem.**	**Hymenoptera**<br>**Andrenidae:** *Macrotera bicolor* Smith, 1853<br>**Apidae:** *Apis mellifera* Linnaeus, 1758; *Diadasia rinconis* Cockerell, 1897<br>**Halictidae:** *Agapostemon texanus* Cresson, 1872<br>**Megachilidae:** *Lithurgus* sp. Berthold, 1827	Mexico	[40]
***Opuntia stricta* Haw.**	**Coleoptera**<br>**Dasytidae:** *Dasytidae* sp. Latreille, 1802<br>**Maloidea:** *Mylabris quadripunctata* Linnaeus, 1767<br>**Mordellidae:** *Mordella* sp. Linnaeus, 1758; *Mordellistena* sp. Costa, 1854<br>**Oedemeridae:** *Oedemera flavipes* Fabricius, 1792<br>**Scarabaeidae:** *Oxythyrea funesta* Poda, 1761<br>**Diptera**<br>**Diptera:** *Diptera* sp. Linnaeus, 1758<br>**Hymenoptera**<br>**Andrenidae:** *Andrena* sp. Fabricius, 1775<br>**Apidae:** *Apis mellifera* Linnaeus, 1758; *Bombus pensylvanicus* De Geer, 1773; *Bombus terrestris* Linnaeus, 1758; *Melissodes* sp. Latreille, 1829; *Mylabris quadripunctata* Linnaeus, 1767; *Xylocopa violacea* Linnaeus, 1758<br>**Colletidae:** *Hylaeus nigritus* Fabricius, 1798<br>**Formicidae:** *Camponotus aethiops* Latreille, 1798<br>**Halictidae:** *Agapostemon splendens* Lepeletier, 1841; *Halictus pyrenaeus* Pérez, 1903; *Lasioglossum* sp. Curtis, 1833<br>**Megachilidae:** *Anthidium* sp. Fabricius, 1804; *Megachile brevis* Say, 1837; *Rhodanthidium sticticum* Fabricius, 1787<br>**Scoliidae:** *Campsomeris quadrimaculata* Fabricius, 1775; *Scolia* sp. Fabricius, 1775<br>**Lepidoptera**<br>*Lepidoptera* sp. Linnaeus, 1758	U.S.A., Spain	[19,55,112]

Opuntia species	Insect species	Country	References
Opuntia sulphurea **Gillies ex Salm-Dyck**	**Hymenoptera**<br>**Andrenidae:** *Arhysosage* sp. Brèthes, 1922<br>**Apidae:** *Apidae* sp. Latreille, 1802; *Bombus morio* Swederus, 1787; *Diadasia patagonica* Brèthes, 1910; *Ptilothrix tricolor* Friese, 1906; *Tetrapedia* sp. Klug, 1810<br>**Megachilidae:** *Lithurgus* sp. Berthold, 1827; *Megachile* sp. Latreille, 1802	Argentin a	[107]
Opuntia tomentosa **Salm-Dyck**	**Coleoptera**<br>**Coleoptera:** *Coleoptera* sp. Linnaeus, 1758<br>**Nitidulidae:** *Nitidulidae* sp. Latreille, 1802<br>**Hymenoptera**<br>**Apidae:** *Bombus* sp. Latreille, 1802<br>**Formicidae:** *Formicidae* sp. Latreille, 1809<br>**Halictidae:** *Lasioglossum* sp. Curtis, 1833<br>**Megachilidae:** *Megachile* sp. Latreille, 1802<br>**Lepidoptera**<br>**Lepidoptera:** *Lepidoptera* sp. Linnaeus, 1758	Mexico	[39]

Opuntia species	Insect species	Country	References
***Opuntia viridirubra* (F.Ritter) P. J. Braun & Esteves**	**Hymenoptera**<br>**Andrenidae:** *Anthrenoides micans* Urban, 1995; *Arhysosage cactorum* Moure, 1999<br>**Apidae:** *Alloscirtetica gilva* Holmberg, 1884; *Ancyloscelis romeroi* Holmberg, 1903; *Apis mellifera* Linnaeus, 1758; *Centris tricolor* Friese, 1900; *Ceratina asuncionis* Strand, 1910; *Ceratina hyemalis* Moure; *Ceratina ischiocentra* Moure; *Chalepogenus goeldianus* Friese, 1899; *Chalepogenus muelleri* Friese, 1899; *Plebeia wittmanni* Moure & Camargo, 1989; *Ptilothrix fructifera* Holmberg, 1903; *Tetrapedia rugulosa* Friese, 1899; *Trigona spinipes* Fabricius, 1793; *Xylocopa augusti* Lepeletier, 1841<br>**Colletidae:** *Cephalocolletes rugata* Urban; *Sarocolletes* sp. Michener, 1989<br>**Halictidae:** *Augochlora amphitrite* Schrottky, 1909; *Augochlora daphnis* Smith, 1853; *Augochlora* sp. **(2)** Smith, 1853; *Augochloropsis euterpe* Holmberg, 1886; *Dialictus* sp. **(2)** Robertson, 1902; *Thectochlora alaris* Vachal, 1904<br>**Megachilidae:** *Lithurgus rufiventris* Friese, 1908; *Megachile guaranitica* Schrottky, 1909; *Megachile* sp. **(2)** Latreille, 1802	Brazil	[59]
