# Supplementary material for: A Systematic Review on Opuntia (Cactaceae; Opuntioideae) Flower-Visiting Insects in the World with Emphasis on Mexico: Implications for Biodiversity Conservation
Source: Plants (Basel). 2022 Jan 4;11(1):131. doi: 10.3390/plants11010131 (PMC8747471; doi:10.3390/plants11010131)
Supplement: Supplementary file 1 [file plants-11-00131-s001.zip › Supplementary Materials Table S2.pdf]

Table S2. List of *Opuntia* and insect species that constitute part of the generalist core in both the global and Mexican networks.

Global network					
**Order**	**Family**	**Insect species**	**Core values (Gc)**	***Opuntia* spp.**	**Core values (Gc)**
Coleoptera	Nitidulidae	*Carpophilus* sp.	1.29	*O. ficus-indica*	3.96
		Nitops pallipennis	1.29	*O. pilifera*	1.8
	Scarabaeidae	*Oxythyrea funesta*	1.29	*O. monacantha*	1.74
Diptera	Diptera	*Diptera* sp.	1.29		
Hymenoptera	Andrenidae	*Arhysosage* sp.	1.29		
		Macrotera bicolor	2.15		
	Apidae	*Apidae* sp.	1.29		
		Apis mellifera	7.28		
		Bombus morio	3		
		Bombus pensylvanicus	1.29		
		Bombus sp.	2.15		
		Bombus terrestris	2.5		
		Ceratina sp.	1.29		
		Diadasia patagonica	2.5		
		Diadasia rinconis	4.72		
		Diadasia sp.	2.15		
		Melissodes sp.	2.15		
		Melissodes tristis	1.29		
		Ptilothrix tricolor	3		
		Xylocopa violacea	1.29		
	Formicidae	*Formicidae* sp.	1.29		
	Halictidae	*Agapostemon* sp.	1.29		
		Agapostemon texanus	4.72		
		Augochlora amphitrite	1.29		
		Augochlora sp.	1.29		
		Lasioglossum sp.	4.72		
	Megachilidae	*Ashmeadiella* sp.	1.29		

		Lithurgus apicalis	1.29		
		Lithurgus littoralis	2.15		
		Lithurgus rufiventris	2.15		
		Lithurgus sp.	4.72		
		Megachile sp.	5.58		
	Vespidae	*Polybia ignobilis*	1.29		
Lepidoptera	Lepidoptera	*Lepidoptera* sp.	3		
**Mexican network**					
**Order**	**Family**	**Insect species**	**Core values (Gc)**	***Opuntia* spp.**	**Core values (Gc)**
Hymenoptera	Andrenidae	*Macrotera bicolor*	3.4	*O. pilifera*	2.36
	Apidae	*Apis mellifera*	3.4		
		Diadasia rinconis	2.11		
		Diadasia sp.	3.4		
	Halictidae	*Agapostemon texanus*	2.11		
		Lasioglossum sp.	2.11		
	Megachilidae	*Ashmeadiella* sp.	2.11		
		Lithurgus littoralis	3.4		
		Megachile sp.	3.4		
