# Supplementary figures and images for: A Systematic Review on Opuntia (Cactaceae; Opuntioideae) Flower-Visiting Insects in the World with Emphasis on Mexico: Implications for Biodiversity Conservation
Source: Plants (Basel). 2022 Jan 4;11(1):131. doi: 10.3390/plants11010131 (PMC8747471; doi:10.3390/plants11010131)

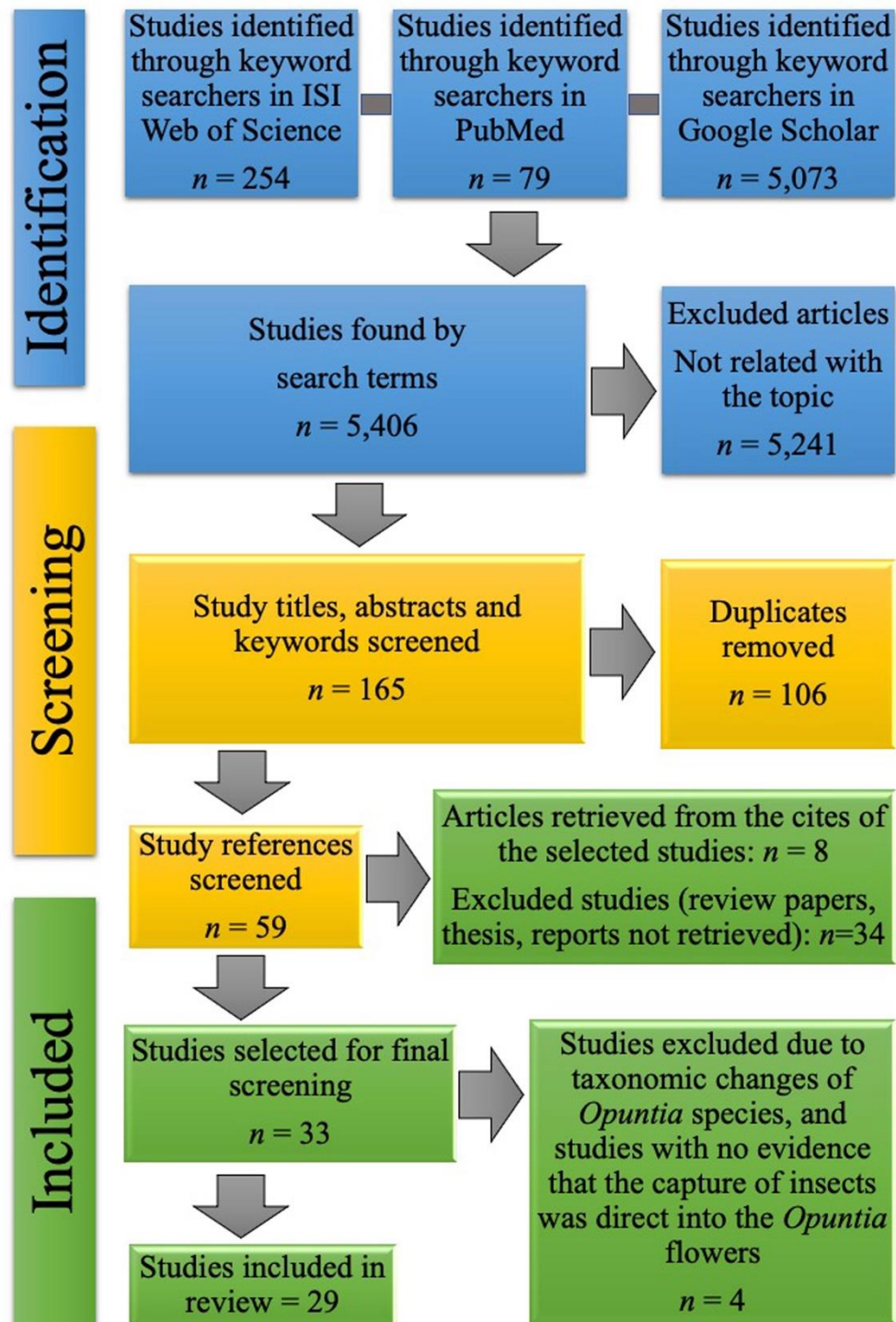

**Figure S1.** Selection of studies for inclusion in the systematic review ( $n$ : the number of studies).

Supplement: Supplementary file 1 [file plants-11-00131-s001.zip › Supplementary Material Figure S1.pdf]
